# Pure PEDOT:PSS hydrogels

**DOI:** 10.1038/s41467-019-09003-5

**Published:** 2019-03-05

**Authors:** Baoyang Lu, Hyunwoo Yuk, Shaoting Lin, Nannan Jian, Kai Qu, Jingkun Xu, Xuanhe Zhao

**Affiliations:** 1 School of Pharmacy, Jiangxi Science and Technology Normal University, 330013 Nanchang, China; 2 Department of Mechanical Engineering, Massachusetts Institute of Technology, Cambridge, MA 02139 USA; 3 School of Chemistry and Molecular Engineering, Qingdao University of Science and Technology, 266042 Qingdao, China; 4 Department of Civil and Environmental Engineering, Massachusetts Institute of Technology, Cambridge, MA 02139 USA

## Abstract

Hydrogels of conducting polymers, particularly poly(3,4-ethylenedioxythiophene):poly(styrene sulfonate) (PEDOT:PSS), provide a promising electrical interface with biological tissues for sensing and stimulation, owing to their favorable electrical and mechanical properties. While existing methods mostly blend PEDOT:PSS with other compositions such as non-conductive polymers, the blending can compromise resultant hydrogels’ mechanical and/or electrical properties. Here, we show that designing interconnected networks of PEDOT:PSS nanofibrils via a simple method can yield high-performance pure PEDOT:PSS hydrogels. The method involves mixing volatile additive dimethyl sulfoxide (DMSO) into aqueous PEDOT:PSS solutions followed by controlled dry-annealing and rehydration. The resultant hydrogels exhibit a set of properties highly desirable for bioelectronic applications, including high electrical conductivity (~20 S cm^−1^ in PBS, ~40 S cm^−1^ in deionized water), high stretchability (> 35% strain), low Young’s modulus (~2 MPa), superior mechanical, electrical and electrochemical stability, and tunable isotropic/anisotropic swelling in wet physiological environments.

## Abstract (web summary)

Hydrogels of conducting polymers provide an electrical interface with biological tissues for sensing and stimulation, but currently have compromised mechanical and electrical properties. Here, the authors show a simple method to achieve pure PEDOT:PSS hydrogels that exhibit superior mechanical and electrical properties, stability, and tunable swelling.

## Introduction

Recent advances in bioelectronics are making the gap between electronic systems and human body ever closer^1–5^. A number of bioelectronic devices, such as commercially available silicon probes^3^, epidermal electronics^6^, stretchable neural interfaces^7–9^, and nanoscale sensor arrays^10^, have shown a great promise toward seamless merging of biology and electronics. Despite these recent successes, the majority of bioelectronic devices still rely on electrode materials, which are physically and mechanically dissimilar to biological tissues. Biological tissues are typically soft (e.g., Young’s moduli in the range of 1 kPa–1 MPa) and contain large amounts of water (e.g., over 70%) with dissolved ionic species^1,3^. In contrast, most inorganic materials (e.g., Si, Au, Pt, and Sn) and dry polymers (e.g., polycarbonate and polyimide) in bioelectronic devices exhibit much higher elastic moduli (e.g., Young’s moduli in the range of 100 MPa–10 GPa) with virtually no water content^1,2^. Hence, the search toward more tissue-like bioelectronic interface has been a grand but ongoing challenge in the field of bioelectronics.

Among many engineering materials, hydrogels show a great promise as ideal interfacing materials to biological tissues, owing to their unique tissue-like mechanical property, water-rich nature, superior biocompatibility, and flexibility and versatility in designing their properties^11–13^. However, conventional hydrogels typically lack electronic conductivity, and the ionic conductivity of hydrogels in physiological conditions is very low (e.g., 6–9 orders of magnitude lower than the conductivity of metals)^14^. Unlike conventional hydrogels, conducting polymer hydrogels offer both electronic and ionic conductivity, rendering them as one of the most promising materials in the emerging field of hydrogel bioelectronics^15–18^. Particularly, poly(3,4-ethylenedioxythiophene):poly(styrene sulfonate) (PEDOT:PSS) based hydrogels have attracted extensive studies owing to their favorable cytocompatibility^19,20^. Existing methods to prepare PEDOT:PSS hydrogels mostly rely on mixing or in situ polymerization of PEDOT:PSS within non-conductive hydrogel templates to form interpenetrating polymer networks (IPN)^15,18,21–23^. However, such IPN-based conducting polymer hydrogels can potentially compromise electrical conductivity and/or electrochemical performances as the non-conductive hydrogel network acts as an electrical insulator (e.g., electrical conductivity is typically below 1 S cm^−1^ in deionized water)^15,18,21,22^. While conductive nano-fillers, such as metal nanoparticles/wires, carbon nanotubes, and graphene, have also been added into IPN-based PEDOT:PSS hydrogels to enhance electrical conductivity^15,24,25^, the dispersion of nano-fillers within polymer chains of hydrogel networks (typically sub-nm scale) can invite potential issues such as inhomogeneity in mechanical and electrical properties, as well as instability and cytotoxicity in contact with wet biological tissues^15,24–27^. In light of these challenges, pure PEDOT:PSS hydrogels have been developed by avoiding the use of other compositions such as non-conducting hydrogel template and/or nano-fillers^28–32^, but they still face numerous technical challenges including low electrical conductivity (<10 S cm^−1^), low stretchability (<10% strain), high Young’s moduli (>100 MPa), and/or poor stability in wet physiological environments due to the absence of supporting matrix. While a recent work reports the highest electrical conductivity (8.8 S cm^−1^) of pure PEDOT:PSS hydrogels^33^, the hydrogel requires concentrated sulfuric acid to fabricate and the electrical conductivity is tested in an acidic solution (pH 1), making it unsuitable for in vivo bioelectronic applications. In addition, the swelling properties of PEDOT:PSS hydrogels in wet physiological environments are of particular importance to their applications in bioelectronics, but such swelling properties have yet been well studied or controlled.

Here, we show that designing interconnected networks of PEDOT:PSS nanofibrils via a simple yet effective method can achieve pure PEDOT:PSS hydrogels with extraordinary electrical, mechanical, and swelling properties without blending other compositions. We show that adding volatile additive dimethyl sulfoxide (DMSO) into aqueous PEDOT:PSS solutions followed by controlled dry-annealing, and subsequent rehydration can yield pure PEDOT:PSS hydrogels, of which electrical, mechanical, and swelling properties can be systematically tuned by the amount of added DMSO and the way of dry-annealing. The pure PEDOT:PSS hydrogels with the optimized amount of DMSO (13 vol.%) and annealing condition (three cycles of 30 min annealing at 130 °C) give superior electrical conductivity of ~20 S cm^−1^ in phosphate buffered saline (PBS) and ~40 S cm^−1^ in deionized water, as well as low Young’s modulus of ~2 MPa and high stretchability of over 35% strain in wet physiological environments. (Note that vol.% indicates the volume fraction of the added DMSO to the final solution volume.) The resultant pure PEDOT:PSS hydrogels exhibit remarkably long-term mechanical (no observable damage over 3 months), electrical (negligible change in electrical conductivity over 3 months), and electrochemical stability (less than 10% change in charge storage capability and charge injection capacity values after 20,000 charging and discharging cycles) in wet physiological conditions. In addition, we discover that the formation of isotropic or anisotropic interconnected networks of PEDOT:PSS nanofibrils by controlled dry-annealing of the PEDOT:PSS solution under mechanically unconstrained or constrained conditions can yield a pure PEDOT:PSS hydrogel that swells isotropically or anisotropically, respectively, providing tunable swelling behaviors compatible to various fabrication approaches. We further demonstrate highly conductive, stretchable, and stable pure PEDOT:PSS hydrogels both in free-standing and robust laminate forms with complex patterns, uniquely enabled by the proposed method.

## Results

### Design of pure PEDOT:PSS hydrogels

**Fig. 1:**
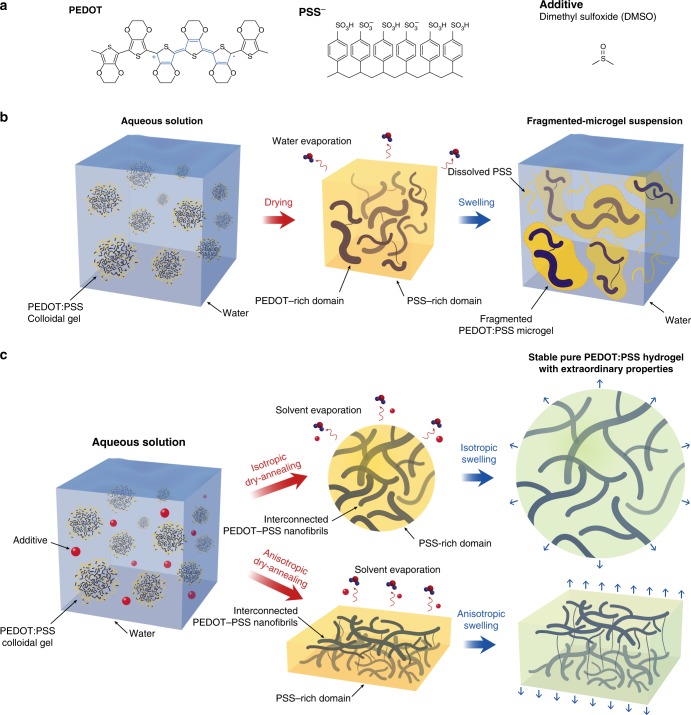
Schematic illustration of pure PEDOT:PSS hydrogel preparation. **a** Chemical structures of PEDOT, PSS, and DMSO. **b** Typical drying and swelling processes of pristine PEDOT:PSS without DMSO. **c** Dry-annealing and swelling processes of PEDOT:PSS with DMSO as the additive. Isotropic dry-annealing of PEDOT:PSS aqueous solution with DMSO leads to isotropic swelling of the stable pure PEDOT:PSS hydrogel with extraordinary properties, while anisotropic dry-annealing provides anisotropic swelling in out-of-plane or thickness direction

Stable water dispersion of PEDOT is generally achieved by introducing anionic polymers such as poly(styrene sulfonic acid) (PSS), which serve as a charge-balancing counter ion template (Fig. 1a). In aqueous colloidal dispersion solution, PEDOT:PSS tends to form micellar microstructures that consist of hydrophobic PEDOT-rich core and hydrophilic PSS-rich shell (Fig. 1b). There are three major molecular interactions in PEDOT:PSS: (i) electrostatic forces of attraction between π-conjugated PEDOT chains and negative charged PSS chains, (ii) π-π stacking of adjacent PEDOT chains, and (iii) interchain entanglements mostly between long PSS chains. To design a pure PEDOT:PSS hydrogel with high electrical conductivity, these molecular interactions should be tamed appropriately to achieve a water-stable network of rigid hydrophobic PEDOT-rich semi-crystalline domains and soft hydrophilic PSS-rich matrix.

One approach to realize pure PEDOT:PSS hydrogels is to dry-anneal the aqueous PEDOT:PSS solution in a controlled manner, and then reswell the dry-annealed PEDOT:PSS into a hydrogel. The loss of water in aqueous PEDOT:PSS solution during the drying process concentrates PEDOT:PSS, and the subsequent annealing in an elevated temperature enables recrystallization of PEDOT-rich nanofibrils and chain rearrangement for both PEDOT and PSS^34–36^. Thus, the resultant dry PEDOT:PSS likely undergoes phase separation into three different domains: (i) rigid conjugated PEDOT-rich crystalline region, (ii) disordered PEDOT:PSS semi-crystalline region, and (iii) PSS-rich soft region^37,38^. By controlling these three domains into a well-distributed network, a stable and highly conductive PEDOT:PSS hydrogel can be achieved by swelling the hydrophilic PSS-rich domains while maintaining the interconnected networks of PEDOT:PSS nanofibrils (Fig. 1c). For this purpose, we choose high boiling point polar co-solvent dimethyl sulfoxide (DMSO) as an additive to the aqueous PEDOT:PSS solution to facilitate the recrystallization of PEDOT-rich nanofibrils and chain rearrangement of PEDOT:PSS during the dry-annealing process^39^. Notably, both solvents (i.e., water and DMSO) can be completely removed by thorough evaporation during the drying (24 h at 60 °C) and the subsequent high-temperature annealing processes (three cycles of 30 min annealing at 130 °C), yielding pure PEDOT:PSS hydrogels as the final product. (See the ‘Method’ section for detailed procedures.)

### Mechanical stability of pure PEDOT:PSS hydrogels

We first dry the pristine aqueous PEDOT:PSS solution without any additive (Fig. 1b). The resultant dried pristine PEDOT:PSS dissociates easily into fragmented microgels in wet environment due to the absence of interconnected PEDOT:PSS nanofibrils to maintain mechanical integrity during the swelling process (Supplementary Fig. 1 and Supplementary Movie 1). The observed instability of the pristine PEDOT:PSS in wet environment is consistent with the previous reports on dissolution of the PEDOT:PSS electrodes in aqueous media^40^.

**Fig. 2:**
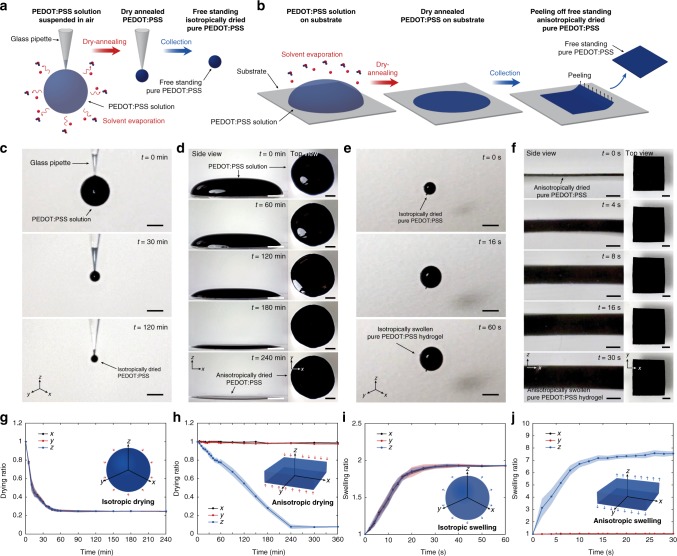
Dry-annealing and swelling behaviors of pure PEDOT:PSS hydrogels. **a** Schematic illustration of isotropic dry-annealing of aqueous PEDOT:PSS solution into a microball. **b** Schematic illustration of anisotropic dry-annealing of aqueous PEDOT:PSS solution into a free-standing film. **c** Experimental images for isotropic dry-annealing of aqueous PEDOT:PSS solution into a microball. **d** Experimental images for anisotropic dry-annealing of aqueous PEDOT:PSS solution into a free-standing film. **e** Isotropic swelling of a pure dry-annealed PEDOT:PSS microball into a stable hydrogel. **f** Anisotropic swelling of a free-standing pure dry-annealed PEDOT:PSS film into a stable hydrogel. **g**, **h** Dimensional changes vs. time during the **g** isotropic and **h** anisotropic dry-annealing of aqueous PEDOT:PSS solution. **i**, **j** Dimensional changes vs. time during the **i** isotropic and **j** anisotropic swelling of pure PEDOT:PSS hydrogels. Values in **g**–**j** represent mean and the error bars represent the SD of the measured values (*n* = 3). Scale bars, 1 mm

Strong polar co-solvents such as DMSO are known to increase the electrical conductivity of PEDOT:PSS by secondary doping^41^ (from ~0.1 to over 10^3^ S cm^−1^). Such conductivity enhancing additives can effectively extend PEDOT:PSS microgel particles from a trapped and/or folded state into linear long chains^39^, and therefore, facilitate the formation of a larger crystalline PEDOT-rich nanofibrils and the interchain entanglements between PSS chains during the drying process^37,39^ (Fig. 1c). By adding 5 vol.% DMSO into the pristine aqueous PEDOT:PSS solution and subsequently dry-annealing (24 h at 60 °C drying followed by three cycles of 30 min annealing at 130 °C) the mixture to thoroughly remove both water and DMSO, we prepare pure PEDOT:PSS microballs or films (Fig. 2a, b and Supplementary Movies 2, 3. See the ‘Method’ section for detailed procedures). The characteristic FT-IR spectral absorption peaks for DMSO (1024 cm^−1^ for stretching vibration of sulfoxyl group; 950 cm^−1^ for bending and 3000 and 2910 cm^−1^ for stretching vibration of methyl group) clearly present in DMSO-containing aqueous PEDOT:PSS solutions while such absorption peaks completely disappear in the FT-IR spectra of the dry-annealed PEDOT:PSS (Supplementary Fig. 2). This indicates that the added DMSO is fully removed during the dry-annealing process, yielding pure PEDOT:PSS.

We further find that the dry-annealed PEDOT:PSS can be facilely converted into stable pure PEDOT:PSS hydrogels by rehydrating in water or in PBS (Fig. 2c–f). The resultant pure PEDOT:PSS hydrogels show high water contents (80–87 wt.% water) and substantially enhanced long-term stability in wet physiological environments (e.g., no observable damage over 3 months in PBS) (Supplementary Fig. 3).

### Swelling properties of pure PEDOT:PSS hydrogels

The swelling behavior of the pure PEDOT:PSS hydrogels is strongly affected by the dry-annealing condition (Fig. 1c). The isotropic dry-annealing of mechanically unconstrained PEDOT:PSS droplet leads to the isotropic swelling behavior (Fig. 2c, e, g, i and Supplementary Movies 2, 4), while the anisotropic drying of mechanically constrained PEDOT:PSS solution (i.e., drying on a substrate) gives the anisotropic swelling behavior over the thickness direction (Fig. 2d, f, h, j and Supplementary Movies 3, 5). The swelling ratio of pure PEDOT:PSS hydrogels in wet environment is also affected by the concentration of DMSO in the aqueous PEDOT:PSS solution. The swelling ratio of pure PEDOT:PSS hydrogels increases with the DMSO concentration used during the preparation up to 20 vol.%, and then the swelling ratio decreases with the DMSO concentration, both in PBS and in deionized water (Supplementary Fig. 4). Furthermore, the pure PEDOT:PSS hydrogels fabricated with the same DMSO concentration exhibit lower swelling ratio in PBS than in deionized water, potentially due to the ionic strength and subsequent change of equilibrium swelling in PBS (Supplementary Fig. 4).

Notably, crosslinkers such as 3-glycidoxypropyltrimethosilane (GOPS) have previously been adopted to achieve stable PEDOT:PSS hydrogels in wet environment as well^32^, but these crosslinked PEDOT:PSS hydrogels exhibit relatively low water contents (<50%) and high Young’s modulus (>100 MPa) with unclear electrical conductivity in the swollen state, significantly limiting their utility in bioelectronic applications.

### Morphologies of dry-annealed PEDOT:PSS

**Fig. 3:**
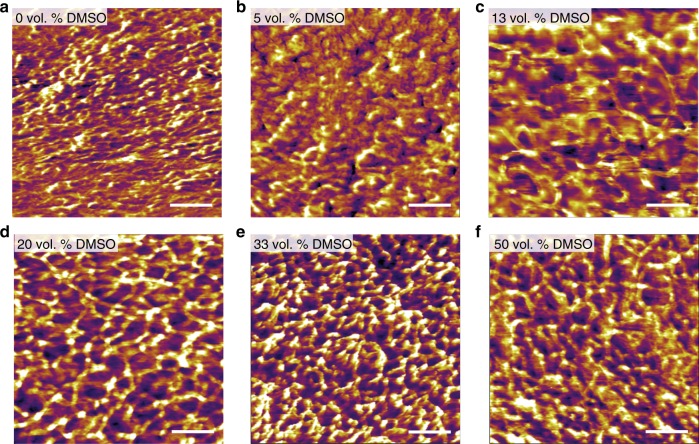
AFM phase images of dry-annealed PEDOT:PSS free-standing films. **a** Pristine PEDOT:PSS film. **b**–**f** Dry-annealed pure PEDOT:PSS films prepared by adding different amount of DMSO by **b** 5 vol.%, **c** 13 vol.%, **d** 20 vol.%, **e** 33 vol.%, and **f** 50 vol.%. Note that vol.% indicates the volume fraction of the added DMSO to the final solution volume. The measured surface roughness is **a** 1.6 nm, **b** 1.4 nm, **c** 2.5 nm, **d** 2.0 nm, **e** 2.1 nm, and **f** 3.1 nm. Scale bars, 100 nm

We further investigate the effect of varying DMSO concentrations in aqueous PEDOT:PSS solution on the morphologies of the resultant dry-annealed PEDOT:PSS via AFM phase imaging (Fig. 3). In comparison with the pristine PEDOT:PSS film without adding DMSO (Fig. 3a), the introduction of DMSO (5–50 vol.%) results in the increased phase separation between PEDOT-rich domains (bright color) and PSS-rich domains (dark color), which originates from the enhanced crystallinity of π-stacking of PEDOT chains during the dry-annealing process^41–45^ (Fig. 3b–f). Notably, higher DMSO concentration further facilitates the growth and interconnection of PEDOT-rich domains, and a well-percolated networks of PEDOT:PSS nanofibrils start to appear at DMSO concentration over 13 vol.% (Fig. 3c–e). Such interconnected nanofibrillar morphology can potentially provide improved electrical conductivity and mechanical properties by forming more effective pathways for both electron transfer and sustaining mechanical forces^46^. However, too high DMSO concentration (over 50 vol.%) begins to lead to the aggregation of the fibrous networks of PEDOT-rich domains (Fig. 3f), similar to the instant aggregation or gelation phenomena previously reported in the addition of sulfuric acid and ionic liquids^33,46^.

We also perform wide-angle X-ray scattering (WAXS) tests in order to further study the effect of varying DMSO concentrations on the morphologies of the resultant dry-annealed pure PEDOT:PSS. The WAXS profiles of the dry-annealed pure PEDOT:PSS films based on varying concentrations of DMSO exhibit the increasing intensity of the scattering vector (*q*) peak at ~19 nm^−1^ (characteristic peak for PEDOT crystalline domain) (Supplementary Fig. 5), further revealing the effective enhancement in the crystallization of PEDOT by the proposed method^47,48^.

### Mechanical properties of pure PEDOT:PSS hydrogels

**Fig. 4:**
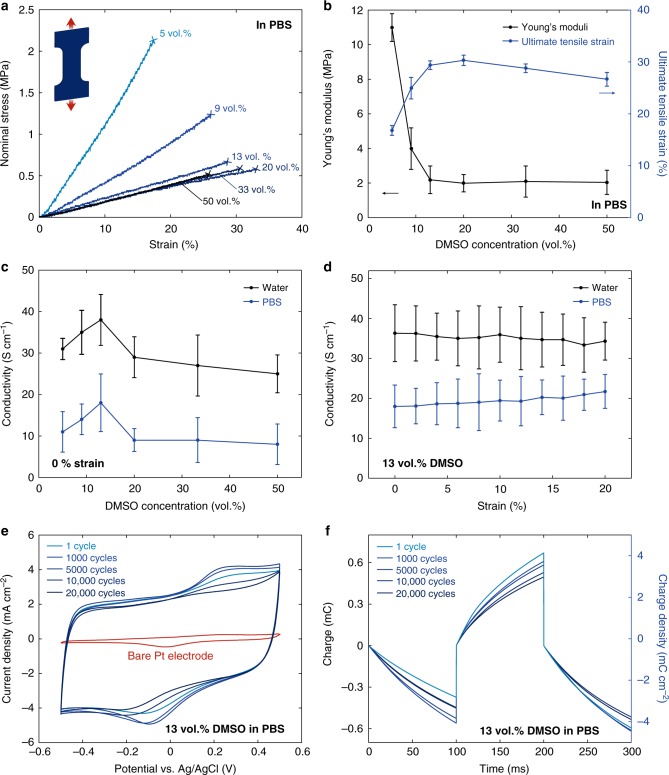
Mechanical and electrical characterizations of pure PEDOT:PSS hydrogels. **a** Nominal stress vs. strain curves of free-standing pure PEDOT:PSS hydrogels in PBS with varying DMSO concentrations. **b** Young’s moduli and ultimate tensile strains vs. DMSO concentration for pure PEDOT:PSS hydrogels in PBS. **c** Electrical conductivity of pure PEDOT:PSS hydrogels both in deionized water and in PBS with varying DMSO concentrations. **d** Electrical conductivity of pure PEDOT:PSS hydrogels at different strain both in deionized water and in PBS. **e** CV curves for the pure PEDOT:PSS hydrogel on Pt electrode in PBS. Red curve represents the CV curve of bare Pt electrode as a control. **f** Cyclic electrochemical current pulse injection curves of the pure PEDOT:PSS hydrogel on Pt electrode under between −1.5 V and 1.5 V vs. Ag/AgCl. Values in **b**–**d** represent mean and the error bars represent the SD of the measured values (*n* = 4)

Taking advantage of the superior stability and free-standing nature of pure PEDOT:PSS hydrogels in wet physiological environments, we systematically characterize the mechanical and electrical properties of free-standing pure PEDOT:PSS hydrogel films both in PBS and in deionized water. Nominal strain vs. stress curves in tensile tests show that the Young’s moduli of pure PEDOT:PSS hydrogels in PBS are in the range of 2–10 MPa (Fig. 4a, b), which are comparable to that of soft elastomers such as polydimethylsiloxane (PDMS) (e.g., Young’s moduli in the range of 1–10 MPa) and several orders of magnitude lower than conventional rigid materials in bioelectronic devices^2,3,15^. The mechanical compliance of pure PEDOT:PSS hydrogels can offer improved long-term biomechanical interactions with biological tissues as demonstrated by PDMS-based bioelectronic implants^7,49,50^. Notably, the pure PEDOT:PSS hydrogels exhibit smaller Young’s moduli in deionized water (in the range of 1–5 MPa) than in PBS (Supplementary Fig. 6), which potentially stems from higher swelling ratio and consequent higher equilibrium water contents of pure PEDOT:PSS hydrogels in deionized water than in PBS (~87 wt.% in deionized water vs. ~80 wt.% in PBS) (Supplementary Fig. 4).

The pure PEDOT:PSS hydrogels show a gradual reduction in Young’s moduli with the increase of DMSO concentration up to 13 vol.% and then keep nearly constant for higher DMSO concentrations, both in PBS and in deionized water (Fig. 4b and Supplementary Fig. 6). The stretchability of the pure PEDOT:PSS hydrogels increases with DMSO concentration up to 20 vol.% and then decreases for higher DMSO concentrations (Fig. 4b and Supplementary Fig. 6). The ultimate tensile strain of the pure PEDOT:PSS hydrogels (20 vol. % DMSO) can reach over 35% in PBS and over 40% in deionized water (Supplementary Figs. 7, 8 and Supplementary Movie 6), which are significantly higher than the dry-annealed pure PEDOT:PSS film before swelling (<10% strain) and closely match the stretchability of biological tissues such as neural tissues (~20% strain)^3^ and skin (~50% strain)^51^. This enhanced stretchability of the pure PEDOT:PSS hydrogels can be attributed to the phase separation of PEDOT and PSS during the dry-annealing process and the resultant interconnected networks of PEDOT:PSS nanofibrils in hydrogel, which is in good agreement with the morphological evolution observed in the AFM phase images (Fig. 3).

### Electrical conductivity of pure PEDOT:PSS hydrogels

We vary the DMSO concentration in the aqueous PEDOT:PSS solution in the range of 5–50 vol.% and keep the same dry-annealing condition of 24 h at 60 °C drying followed by three cycles of 30 min annealing at 130 °C to make a batch of pure PEDOT:PSS hydrogels. During rehydration, the dry-annealed pure PEDOT:PSS absorbs water and ions, significantly decreasing its electrical conductivity from ~500 S cm^−1^ in the dry state to less than 50 S cm^−1^ in the swollen state (both in PBS and in deionized water) (Fig. 4c).

The concentration of DMSO plays an important role in the electrical conductivity of the pure PEDOT:PSS hydrogels. The electrical conductivity and DMSO concentration display a non-monotonic relationship with the highest conductivity of ~20 S cm^−1^ in PBS and ~40 S cm^−1^ in deionized water achieved at 13 vol.% DMSO concentration. The addition of an optimal amount of DMSO (i.e., 13 vol.%) in the aqueous PEDOT:PSS solution allows more preferred formation of better interconnected networks of PEDOT:PSS nanofibrils, which can offer enhanced conducting pathways against the fast swelling of hydrophilic PSS-rich matrix. The reduction in the electrical conductivity with DMSO concentration over 13 vol.% can possibly be attributed to an unfavorable aggregation of the nanofibrillar PEDOT:PSS networks by the presence of excessive amount of DMSO^46^ (Figs. 3c and 4).

The same pure PEDOT:PSS hydrogel also exhibits a reduced electrical conductivity in physiological condition (i.e., in PBS) compared to in deionized water (Fig. 4c). Typical aqueous PEDOT:PSS solutions and pure PEDOT:PSS hydrogels in deionized water are acidic (pH 1–2) due to the presence of PSS. Physiologically-relevant environment such as PBS can neutralize pure PEDOT:PSS hydrogels and such neuralization of acidic PEDOT:PSS has been reported to substantially decrease electrical conductivity by the disruption of the π-π stacking of PEDOT crystalline domain and the consequent reduction in bipolaron concentration (i.e., charge carrier density)^39,52^. We find that the electrical conductivity of pure PEDOT:PSS hydrogels in PBS adjusted to pH 1 recovers high electrical conductivity measured in deionized water (~40 S cm^−1^) (Supplementary Fig. 9), which is consistent with the previous reports^39,52^. It should be noted that, despite the reduced electrical conductivity in PBS, the optimized electrical conductivity of ~20 S cm^−1^ in PBS (~40 S cm^−1^ in deionized water) for the pure PEDOT:PSS hydrogels are among the highest values for conducting polymer hydrogels in PBS and water (Supplementary Table 1)^15,53^. Furthermore, the electrical conductivity of pure PEDOT:PSS hydrogels exhibit superior stability in wet environments with negligible decrease over 3 months both in PBS and in deionized water (Supplementary Fig. 10a). Such high and stable electrical conductivity in wet physiological environments can ensure more efficient and reliable bioelectronic stimulation and recoding, and therefore, will be highly advantageous for bioelectronic devices and applications^15^.

Upon tensile deformation, the pure PEDOT:PSS hydrogels in deionized water display a slight reduction in conductivity similar to the previously reported stretchable PEDOT:PSS films^46^ (Fig. 4d and Supplementary Fig. 11). Interestingly, tensile deformation of the pure PEDOT:PSS hydrogels in PBS can slightly increase its electrical conductivity (Supplementary Fig. 12), whose mechanism will be studied in the future work.

### Charge storage and injection properties of pure PEDOT:PSS hydrogels

We further characterize other electrical properties such as charge storage capability (CSC) and charge injection capacity (CIC) of pure PEDOT:PSS hydrogels to evaluate their performance in bioelectronic applications. Cyclic voltammetry (CV) of the pure PEDOT:PSS hydrogels measured on Pt electrode demonstrate that pure PEDOT:PSS hydrogels (13 vol.% DMSO concentration) show a high CSC value of 60 mC cm^−2^ and superior electrochemical stability against charging and discharging cycles in wet physiological environment (i.e., PBS) with less than 9% reduction in CSC after 20,000 CV cycles (Fig. 4e and Supplementary Fig. 10b). The pure PEDOT:PSS hydrogels (13 vol.% DMSO concentration) also exhibit high CIC value of 8.3 mC cm^−2^ and stability (less than 10% change after 20,000 cycles) in PBS (Fig. 4f and Supplementary Fig. 10c). These high CSC and CIC values of pure PEDOT:PSS hydrogels together with their soft and wet properties are particularly desirable for bioelectronic stimulation applications^15,54^, while their performance as current collectors and electrical interconnects can potentially be further improved by combinatorial use together with highly conductive metallic nanocomposites such as silver nanowires (AgNW)^55,56^.

### Patterning of pure PEDOT:PSS hydrogels

Patterning of electrodes into complex geometries is a crucial step for fabricating bioelectronic devices^6,46,57^. Complicated preparation steps and/or poor stability in aqueous conditions have significantly limited the realization of facile patterning of conductive hydrogels with complex designs^21,33^ (Supplementary Table 1). Our simple but highly effective method enables us to fabricate highly conductive, stable, and stretchable hydrogel patterns. Moreover, tunable swelling behaviors of the pure PEDOT:PSS hydrogels further provide superior compatibility with various fabrication processes by minimizing geometric distortion and interfacial delamination from the substrate in wet environment.

**Fig. 5:**
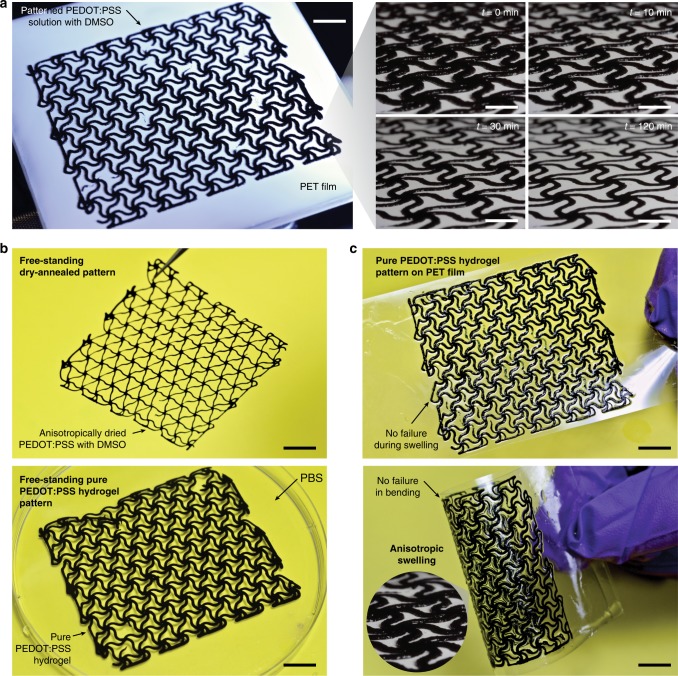
Patterning of pure PEDOT:PSS hydrogels. **a** Patterned PEDOT:PSS solution on PET substrate. Experimental images show anisotropic drying process of the patterned ink over time. **b** Free-standing pure PEDOT:PSS hydrogel pattern can be fabricated by peeling the dry-annealed pattern and swelling in PBS. **c** Robust laminate of pure PEDOT:PSS hydrogel pattern can be fabricated by suppressing delamination via anisotropic swelling behavior. Scale bars, 10 mm (**a**, left panel); 5 mm (**a**, right panel); 8 mm (**b**, **c**)

To demonstrate the capability to pattern complex geometry, a wavy mesh is fabricated by printing the aqueous PEDOT:PSS solution with added DMSO onto a substrate with the resolution of 400 µm (Fig. 5a). After printing, the printed patterns are dry-annealed anisotropically under the mechanical constraint of the substrate (Fig. 5a and Supplementary Movie 7). The dry-annealed patterns can be readily converted into pure PEDOT:PSS hydrogels by rehydrating in wet environments (Fig. 5b,c). The pure PEDOT:PSS hydrogel patterns can be prepared either into free-standing structures by peeling off from low-adhesion substrates (e.g., polypropylene and polydimethylsiloxane) (Fig. 5b), or robust laminates by patterning on high-adhesion substrates (e.g., polyethylene terephthalate) (Fig. 5c). The anisotropic swelling of the hydrogel (resulted from mechanically constrained dry-annealing process) greatly benefits the fabrication of conductive hydrogel patterns by avoiding undesirable dimensional changes in free-standing structures (Fig. 5b) and interfacial failures in laminates (Fig. 5c), which can be advantageous for various fabrication techniques, such as direct ink writing, inkjet printing, and spin coating^46,58^.

## Discussion

In this study, we show that designing interconnected networks of PEDOT:PSS nanofibrils via a simple yet highly effective strategy can realize high-performance pure PEDOT:PSS hydrogels with electrical conductivity as high as 20 S cm^−1^ in PBS (40 S cm^−1^ in deionized water), high stretchability (>35% strain) with low Young’s modulus (~2 MPa), and superior mechanical, electrical, and electrochemical stability in wet physiological environments. The swelling behavior of the pure PEDOT:PSS hydrogels can be tuned by mechanical constraints during the dry-annealing process and the consequent anisotropy in interconnected networks of PEDOT:PSS nanofibrils, providing additional flexibility in their processing and bioelectronic device fabrication. We further demonstrate that the proposed method can realize a facile patterning of stable conductive hydrogels into complex geometries and various form factors. The current study not only addresses a long-lasting challenge in the development of high-performance conducting polymer hydrogels, but also offers a promising new avenue toward next-generation hydrogel bioelectronic devices and applications.

## Methods

### Preparation of pure PEDOT:PSS hydrogel

PEDOT:PSS aqueous solution (1.1–1.3% solid content, Clevios™ PH1000, Heraeus Electronic Materials) was stirred vigorously for 6 h, and then dimethyl sulfoxide (DMSO, Sigma–Aldrich) was added in the range of 0–50 vol.% of the final solution. Upon further stirring for 24 h at room temperature, the mixed solution was solvent-casted directly onto polypropylene (PP) or polyethylene terephthalate (PET) substrate and dried at 60 °C for 24 h followed by multiple cycles of annealing at 130 °C (three cycles with 30 min per each cycle) to yield pure PEDOT:PSS films. Free-standing pure PEDOT:PSS films were obtained by peeling off the dry-annealed samples from PP substrate. To prepare pure PEDOT:PSS microballs, a droplet of the mixed solution was suspended at the tip of a micropipette, and dry-annealed similar to the film samples. To investigate the swelling behavior, dry-annealed pure PEDOT:PSS free-standing films or microballs were immersed into deionized water or PBS (Sigma–Aldrich).

### FT-IR characterization

Free-standing dry-annealed and swollen pure PEDOT:PSS hydrogel films were prepared and used to characterize the infrared spectra by a Fourier-transform infrared (FT-IR) spectrometer (Vertex 70, Bruker). For aqueous PEDOT:PSS solutions, a KBr pellet was employed as a substrate.

### AFM phase imaging

AFM phase images and surface roughness data were acquired by atomic force microscope (MFP-3D, Asylum Research). Dry-annealed free-standing PEDOT:PSS films were directly attached onto sample stage by double-sided carbon tape.

### Wide-angle X-ray scattering (WAXS) characterization

Transmission WAXS measurements were carried out by using a SAXSLAB instrument at the MIT Center for Materials Science and Engineering (CMSE).

### Electrical conductivity measurement

Electrical conductivity for all the samples was measured by using a standard four-point probe (Keithley 2700 digital multimeter, Keithley). Dry-annealed pure PEDOT:PSS films or hydrogels were cut into rectangle shape (30 mm in length and 5 mm in width). Copper wire electrodes (diameter, 0.5 mm) were attached onto the surface of dry-annealed films by applying silver paste, while platinum wire electrodes (diameter, 0.5 mm) were employed for hydrogels to avoid the corrosion in wet environments. For pure PEDOT:PSS hydrogels, a humidifier was employed to keep the samples hydrated throughout all experiments. For the conductivity stability tests, the pure PEDOT:PSS hydrogels were immersed in deionized water or in PBS and tested at different time points (1 day, 1 week, 1 month, 2 months, and 3 months).

### Mechanical characterization

All samples for mechanical characterizations were performed by using fully swollen pure PEDOT:PSS free-standing films with dog-bone shape either in deionized water or in PBS. Tensile property of the samples was measured by a mechanical testing machine (U-Stretch with 4.4 N load cell, CellScale). All mechanical characterizations were performed within the submersion stage filled with either deionized water or PBS to avoid dehydration of the hydrogels.

### Cyclic voltammetry

Cyclic voltammetry of pure PEDOT:PSS hydrogels was performed by using a potentiostat/galvanostat (VersaSTAT 3, Princeton Applied Research). Pt wires (diameter, 1 mm) were employed as both working and counter electrodes, and Ag/AgCl electrode was used as the reference electrode. Prior to all measurements, the electrodes were cleaned successively with abrasive paper, deionized water, and ethyl alcohol. PBS was used as the supporting electrolyte. CSC was calculated from the measured CV curves as

1 $${\mathrm{CSC}} = \mathop {\smallint }\limits_{E_2}^{E_1} \frac{{i\left( E \right)}}{{2vA}}{\mathrm{d}}E$$

where *v* is the scan rate, *E*_2_ − *E*_1_ is the potential window, *i* is the current at each potential, and *A* is the area of the pure PEDOT:PSS hydrogel film.

In order to characterize charge injection performance of pure PEDOT:PSS hydrogels, electrochemical current pulse injection (in recurrent potential pulses mode, −1.5 ~1.5 V vs. Ag/AgCl) tests were performed in PBS by using a electrochemical workstation (VersaSTAT 3, Ametek Scientific Instruments). Ag/AgCl electrode was employed as a reference electrode, platinum wires (diameter, 1 mm) as counter and working electrodes. CIC was calculated from the measured charge injection curves as

2 $${\mathrm{CIC}} = \frac{{Q_{\mathrm{{inj}}({\mathrm{c}})} + Q_{\mathrm{{inj}}({\mathrm{a}})}}}{A}$$

where CIC represents the charge density of pure PEDOT:PSS hydrogel between the reduction potential (cathodal limit) and the oxidation potential (anodal limit), *Q*_inj(c)_ is the total delivered (or injected) charge in cathodal phase, *Q*_inj(a)_ is the total delivered (or injected) charge in anodal phase, and *A* is the area of the pure PEDOT:PSS hydrogel, respectively.

## Supplementary information

Supplementary Information

Description of Additional Supplementary Files

Supplementary Movie 1

Supplementary Movie 2

Supplementary Movie 3

Supplementary Movie 4

Supplementary Movie 5

Supplementary Movie 6

Supplementary Movie 7

## Data Availability

The data that support the findings of this study are available from the corresponding author upon request.

## References

[1] Rivnay J, Wang H, Fenno L, Deisseroth K, Malliaras GG (2017). Next-generation probes, particles, and proteins for neural interfacing. Sci. Adv..

[2] Jeong JW (2015). Soft materials in neuroengineering for hard problems in neuroscience. Neuron.

[3] Lacour SP, Courtine G, Guck J (2016). Materials and technologies for soft implantable neuroprostheses. Nat. Rev. Mater..

[4] Chen R, Canales A, Anikeeva P (2017). Neural recording and modulation technologies. Nat. Rev. Mater..

[5] Someya T, Bao Z, Malliaras GG (2016). The rise of plastic bioelectronics. Nature.

[6] Kim DH (2011). Epidermal electronics. Science.

[7] Minev IR (2015). Electronic dura mater for long-term multimodal neural interfaces. Science.

[8] Tybrandt K (2018). High‐density stretchable electrode grids for chronic neural recording. Adv. Mater..

[9] Qi D (2017). Highly stretchable, compliant, polymeric microelectrode arrays for in vivo electrophysiological interfacing. Adv. Mater..

[10] Xie C (2015). Three-dimensional macroporous nanoelectronic networks as minimally invasive brain probes. Nat. Mater..

[11] Lee KY, Mooney DJ (2001). Hydrogels for tissue engineering. Chem. Rev..

[12] Zhang YS, Khademhosseini A (2017). Advances in engineering hydrogels. Science.

[13] Yuk H, Zhang T, Lin S, Parada GA, Zhao X (2016). Tough bonding of hydrogels to diverse non-porous surfaces. Nat. Mater..

[14] Keplinger C (2013). Stretchable, transparent, ionic conductors. Science.

[15] Yuk, H., Lu, B. & Zhao, X. Hydrogel bioelectronics. *Chem. Soc. Rev.*10.1039/C8CS00595H (2018). 10.1039/c8cs00595h 30474663

[16] Mawad D (2016). A conducting polymer with enhanced electronic stability applied in cardiac models. Sci. Adv..

[17] Groenendaal L, Jonas F, Freitag D, Pielartzik H, Reynolds JR (2000). Poly (3, 4‐ethylenedioxythiophene) and its derivatives: past, present, and future. Adv. Mater..

[18] Feig VR, Tran H, Lee M, Bao Z (2018). Mechanically tunable conductive interpenetrating network hydrogels that mimic the elastic moduli of biological tissue. Nat. Commun..

[19] Venkatraman S (2011). In vitro and in vivo evaluation of PEDOT microelectrodes for neural stimulation and recording. IEEE Trans. Neural Syst. Rehabil. Eng..

[20] Berggren M, Richter‐Dahlfors A (2007). Organic bioelectronics. Adv. Mater..

[21] Lee YY (2016). A strain‐insensitive stretchable electronic conductor: PEDOT: PSS/acrylamide organogels. Adv. Mater..

[22] Warren H, Panhuis Mih (2013). Electrically conducting PEDOT:PSS—gellan gum hydrogels. MRS Proc..

[23] Goding J, Gilmour A, Martens P, Poole‐Warren L, Green R (2017). Interpenetrating conducting hydrogel materials for neural interfacing electrodes. Adv. Healthc. Mater..

[24] Gaharwar AK, Peppas NA, Khademhosseini A (2014). Nanocomposite hydrogels for biomedical applications. Biotechnol. Bioeng..

[25] Choi, S., Han, S. I., Kim, D., Hyeon, T. & Kim, D.-H. High-performance stretchable conductive nanocomposites: materials, processes, and device applications. *Chem. Soc. Rev.*10.1039/C8CS00706C (2018). 10.1039/c8cs00706c 30519703

[26] Smart S, Cassady A, Lu G, Martin D (2006). The biocompatibility of carbon nanotubes. Carbon.

[27] Hussain, M., Kabir, M. & Sood, A. On the cytotoxicity of carbon nanotubes. *Curr. Sci*. **96**, 664–673 (2009).

[28] Mano N, Yoo JE, Tarver J, Loo YL, Heller A (2007). An electron-conducting cross-linked polyaniline-based redox hydrogel, formed in one step at pH 7.2, wires glucose oxidase. J. Am. Chem. Soc..

[29] Dai T, Shi Z, Shen C, Wang J, Lu Y (2010). Self-strengthened conducting polymer hydrogels. Synth. Met..

[30] Pan L (2012). Hierarchical nanostructured conducting polymer hydrogel with high electrochemical activity. Proc. Natl Acad. Sci. USA.

[31] Mawad D (2012). A single component conducting polymer hydrogel as a scaffold for tissue engineering. Adv. Funct. Mater..

[32] ElMahmoudy, M. et al. Tailoring the electrochemical and mechanical properties of PEDOT: PSS films for bioelectronics. *Macromol. Mater. Eng.* **302**, 1600497 (2017).

[33] Yao B (2017). Ultrahigh‐conductivity polymer hydrogels with arbitrary structures. Adv. Mater..

[34] Palumbiny CM (2015). The crystallization of PEDOT: PSS polymeric electrodes probed in situ during printing. Adv. Mater..

[35] Duc C, Vlandas A, Malliaras G, Senez V (2016). Wettability of PEDOT: PSS films. Soft Matter.

[36] Duc C, Vlandas A, Malliaras GG, Senez V (2017). Electrowetting on immersed conducting hydrogel. J. Phys. Chem. B.

[37] Lang U, Müller E, Naujoks N, Dual J (2009). Microscopical investigations of PEDOT: PSS thin films. Adv. Funct. Mater..

[38] Rivnay J (2016). Structural control of mixed ionic and electronic transport in conducting polymers. Nat. Commun..

[39] Shi H, Liu C, Jiang Q, Xu J (2015). Effective approaches to improve the electrical conductivity of PEDOT: PSS: a review. Adv. Electron. Mater..

[40] Ouyang L (2017). Enhanced PEDOT adhesion on solid substrates with electrografted P (EDOT-NH2). Sci. Adv..

[41] Nevrela J (2015). Secondary doping in poly (3, 4‐ethylenedioxythiophene): Poly (4‐styrenesulfonate) thin films. . J. Polym. Sci. Part B Polym. Phys..

[42] Lipomi DJ (2012). Electronic properties of transparent conductive films of PEDOT: PSS on stretchable substrates. Chem. Mater..

[43] Kim N (2014). Highly conductive PEDOT: PSS nanofibrils induced by solution‐processed crystallization. Adv. Mater..

[44] Savagatrup S (2015). Plasticization of PEDOT: PSS by common additives for mechanically robust organic solar cells and wearable sensors. Adv. Funct. Mater..

[45] Worfolk BJ (2015). Ultrahigh electrical conductivity in solution-sheared polymeric transparent films. Proc. Natl Acad. Sci. USA.

[46] Wang Y (2017). A highly stretchable, transparent, and conductive polymer. Sci. Adv..

[47] Kim EG, Brédas JL (2008). Electronic evolution of poly (3, 4-ethylenedioxythiophene)(PEDOT): from the isolated chain to the pristine and heavily doped crystals. J. Am. Chem. Soc..

[48] Takano T, Masunaga H, Fujiwara A, Okuzaki H, Sasaki T (2012). PEDOT nanocrystal in highly conductive PEDOT: PSS polymer films. Macromolecules.

[49] Park SI (2015). Soft, stretchable, fully implantable miniaturized optoelectronic systems for wireless optogenetics. Nat. Biotechnol..

[50] Jeong JW (2015). Wireless optofluidic systems for programmable in vivo pharmacology and optogenetics. Cell.

[51] Jang KI (2015). Soft network composite materials with deterministic and bio-inspired designs. Nat. Commun..

[52] Mochizuki Y, Horii T, Okuzaki H (2012). Effect of pH on structure and conductivity of PEDOT/PSS. Trans. Mater. Res. Soc. Jpn..

[53] Liu Y (2019). Soft and elastic hydrogel-based microelectronics for localized low-voltage neuromodulation. Nat. Biomed. Eng..

[54] Cogan SF (2008). Neural stimulation and recording electrodes. Annu. Rev. Biomed. Eng..

[55] Park J (2016). Electromechanical cardioplasty using a wrapped elasto-conductive epicardial mesh. Sci. Transl. Med..

[56] Choi S (2018). Highly conductive, stretchable and biocompatible Ag–Au core–sheath nanowire composite for wearable and implantable bioelectronics. Nat. Nanotechnol..

[57] Kleber C, Bruns M, Lienkamp K, Rühe J, Asplund M (2017). An interpenetrating, microstructurable and covalently attached conducting polymer hydrogel for neural interfaces. Acta Biomater..

[58] Sirringhaus H (2000). High-resolution inkjet printing of all-polymer transistor circuits. Science.

