# Supplementary material for: Pure PEDOT:PSS hydrogels
Source: Nat Commun. 2019 Mar 5;10:1043. doi: 10.1038/s41467-019-09003-5 (PMC6401010; doi:10.1038/s41467-019-09003-5)
Supplement: Supplementary file 1 — Supplementary Information [file 41467_2019_9003_MOESM1_ESM.pdf]

Pure PEDOT:PSS Hydrogels

Lu et al.

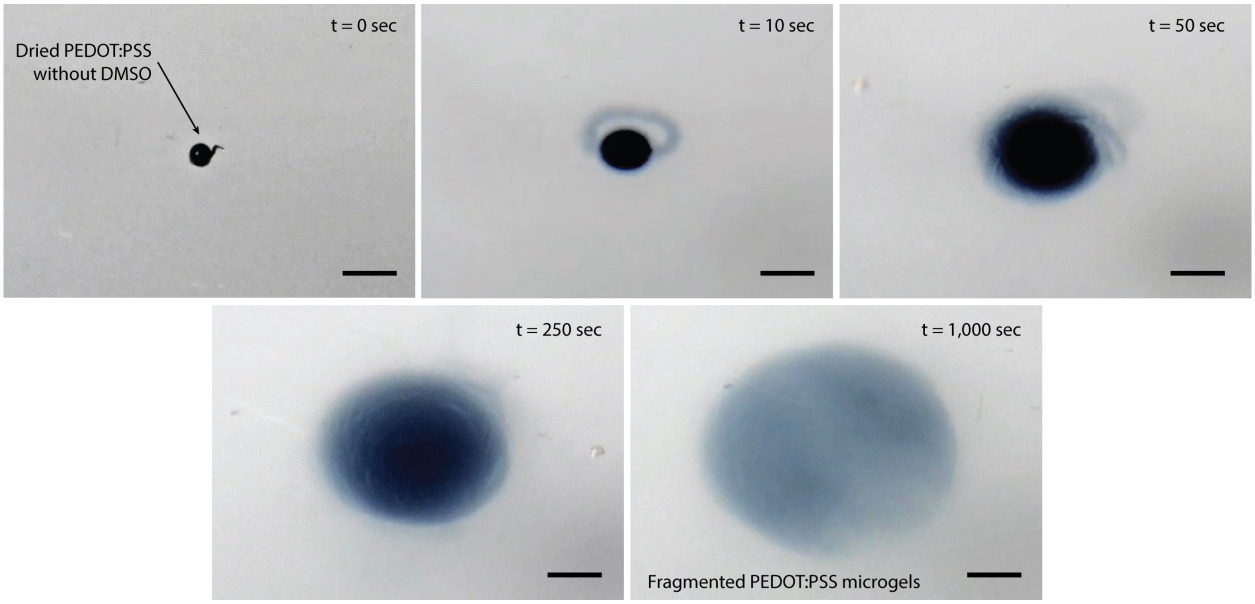

Supplementary Figure 1 | Dissociation of pristine PEDOT:PSS in wet environment. Dried pristine PEDOT:PSS microball swells and readily dissociates into fragmented microgels instead of forming a stable hydrogel. Scale bar, 1 mm.

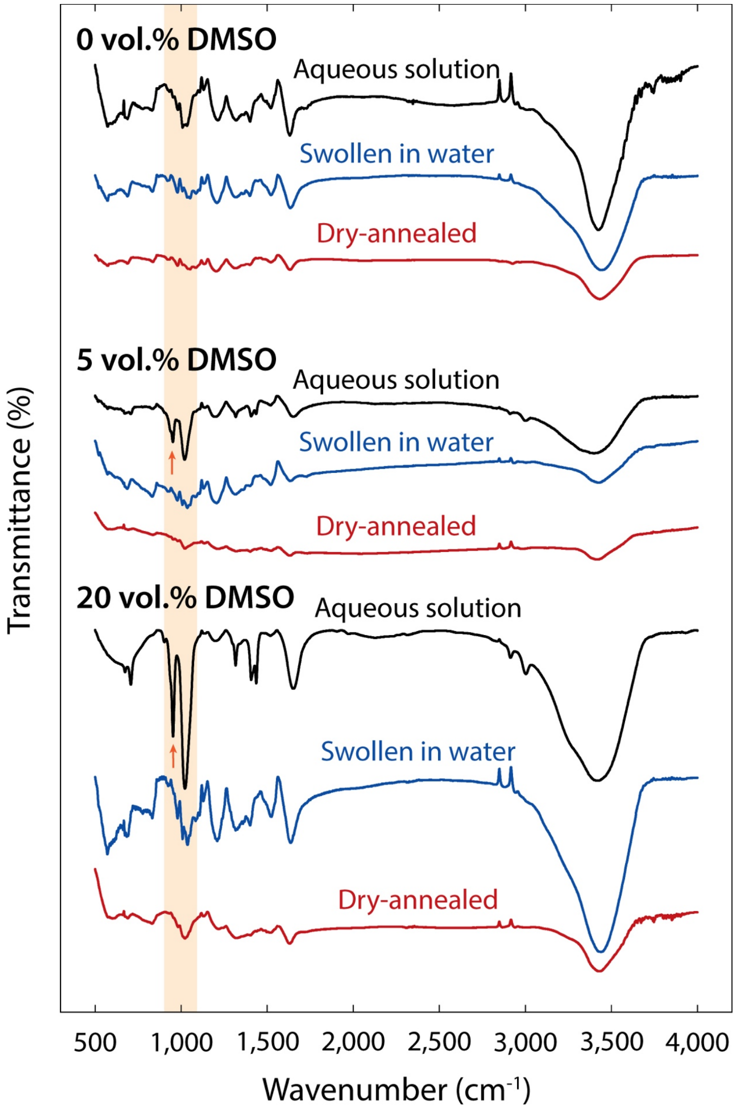

Supplementary Figure 2 | FT-IR spectra of various PEDOT:PSS solutions, dry-annealed and swollen pure PEDOT:PSS hydrogels. The PEDOT:PSS aqueous solutions with varying DMOS concentrations (0, 5, and 20 vol.%) display characteristic absorption peaks for DMSO (1,024 cm^{-1} for stretching vibration of sulfoxyl group; 950 cm^{-1} for bending and 3,000 and 2,910 cm^{-1} for stretching vibration of methyl group), while these peaks for DMSO disappear for dry-annealed and swollen pure PEDOT:PSS hydrogels.

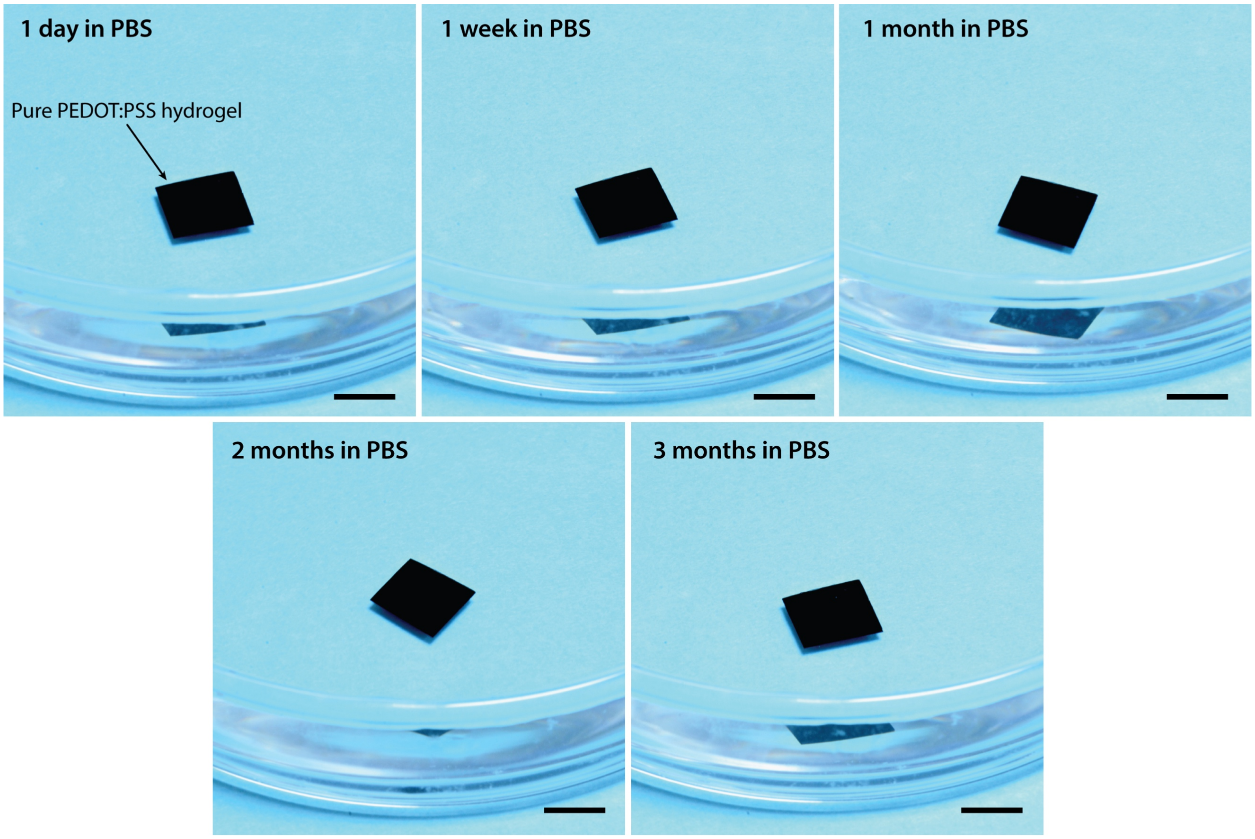

Supplementary Figure 3 | Long-term stability of pure PEDOT:PSS hydrogel in wet physiological environment. Pure PEDOT:PSS hydrogel prepared from the PEDOT:PSS aqueous solution with 13 vol.% DMSO shows extraordinary stability in PBS over 3 months without any visible degradation or dissociation. Scale bar, 10 mm.

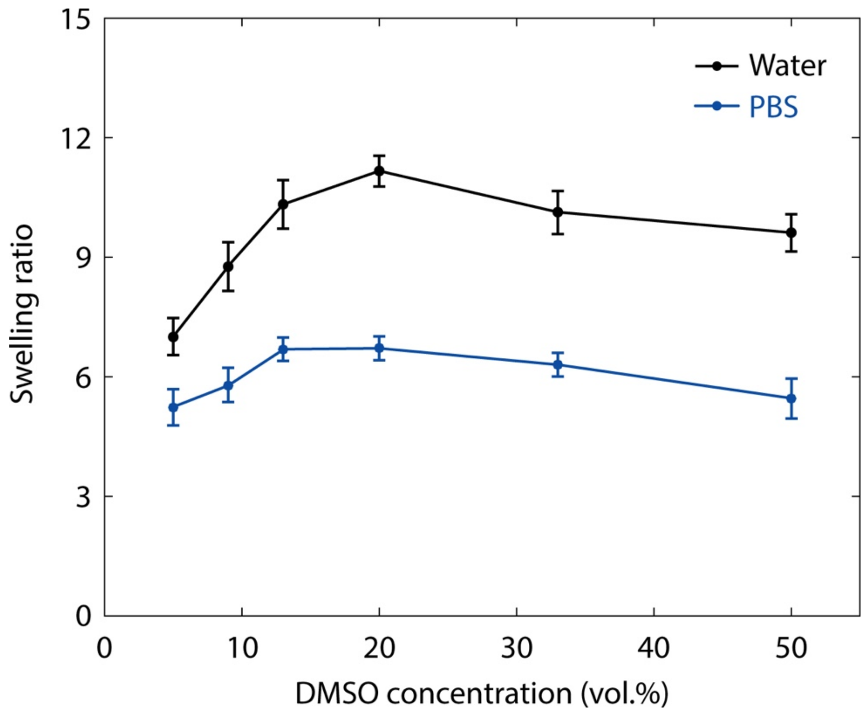

Supplementary Figure 4 | Swelling ratio vs. DMSO concentration of pure PEDOT:PSS hydrogels in wet environments. Swelling ratio of pure PEDOT:PSS hydrogels prepared based on varying DMSO concentrations both in PBS and in deionized water. Values represent mean and the error bars represent the s.d. of measured values (n = 4).

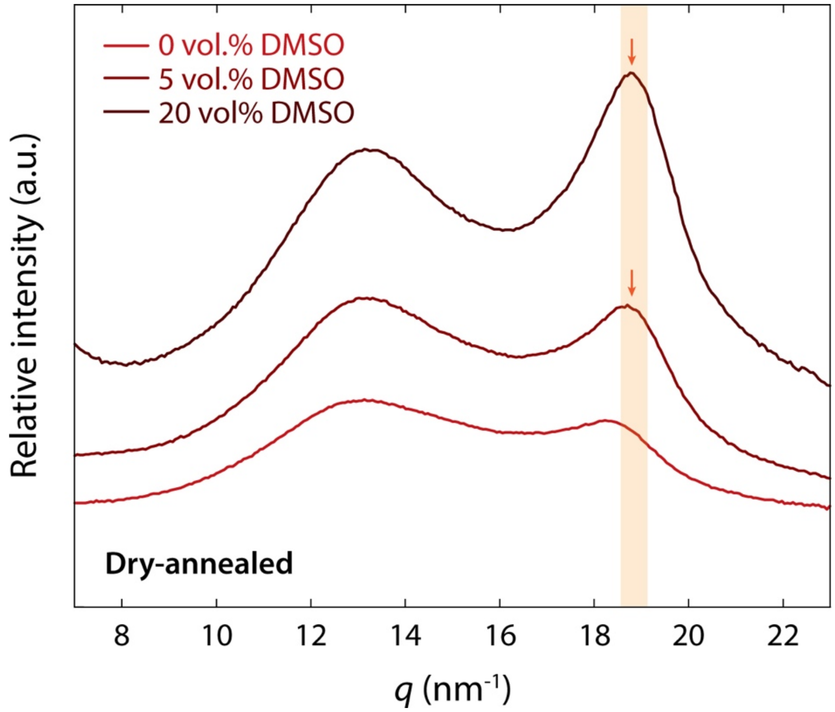

Supplementary Figure 5 | WAXS profiles of dry-annealed and swollen pure PEDOT:PSS hydrogels. The WAXS profiles of dry-annealed pure PEDOT:PSS films based on varying DMSO concentrations (0, 5, and 20 vol.%). The profiles are shifted vertically for clarity.

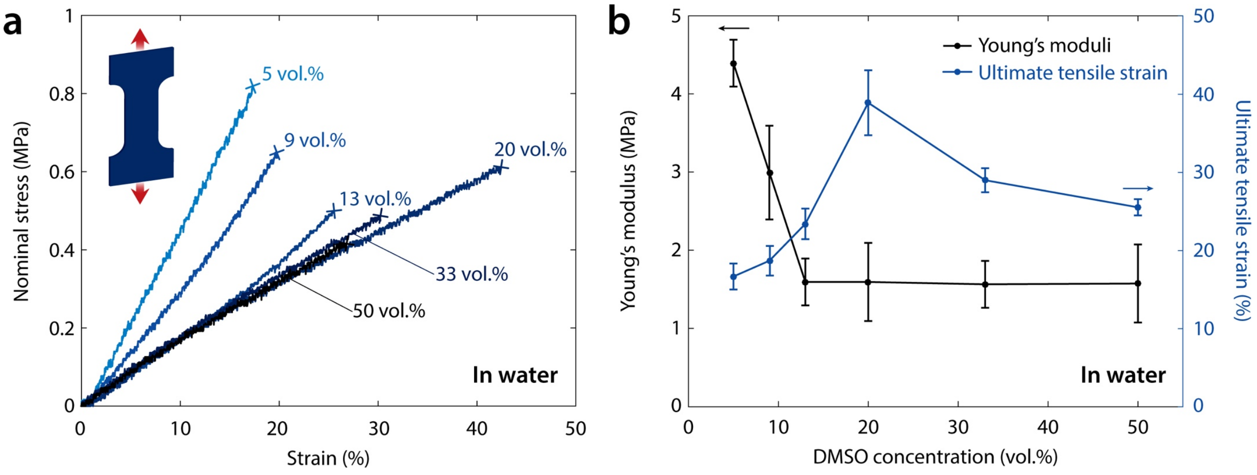

Supplementary Figure 6 | Mechanical characterizations of pure PEDOT:PSS hydrogels in deionized water. (**a**) Nominal stress vs. strain curves of pure PEDOT:PSS hydrogels in deionized water based on varying DMSO concentrations. (**b**) Young's moduli and ultimate tensile strains vs. DMSO concentration for pure PEDOT:PSS hydrogels in deionized water. Values in **b** represent mean and the error bars represent the s.d. of measured values ($n = 4$).

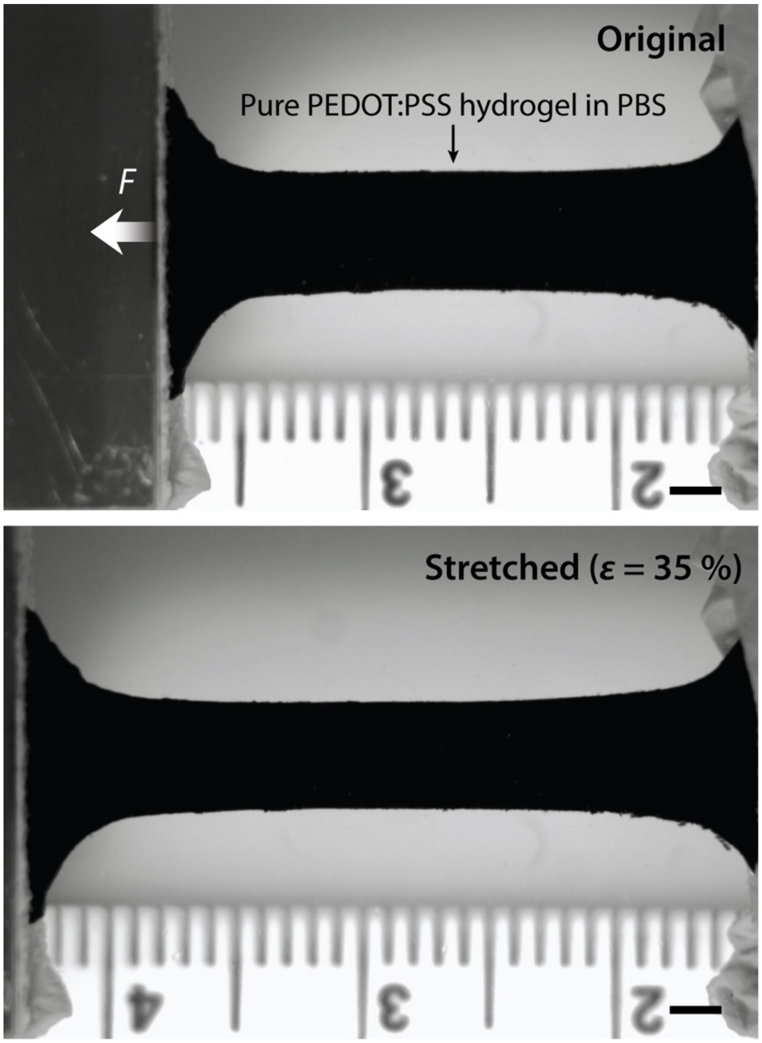

Supplementary Figure 7 | Tensile deformation of pure PEDOT:PSS hydrogel in PBS. Pure PEDOT:PSS hydrogel exhibits good stretchability and can sustain tensile deformation over 35 % in wet physiological environment without failure. Scale bar, 2 mm.

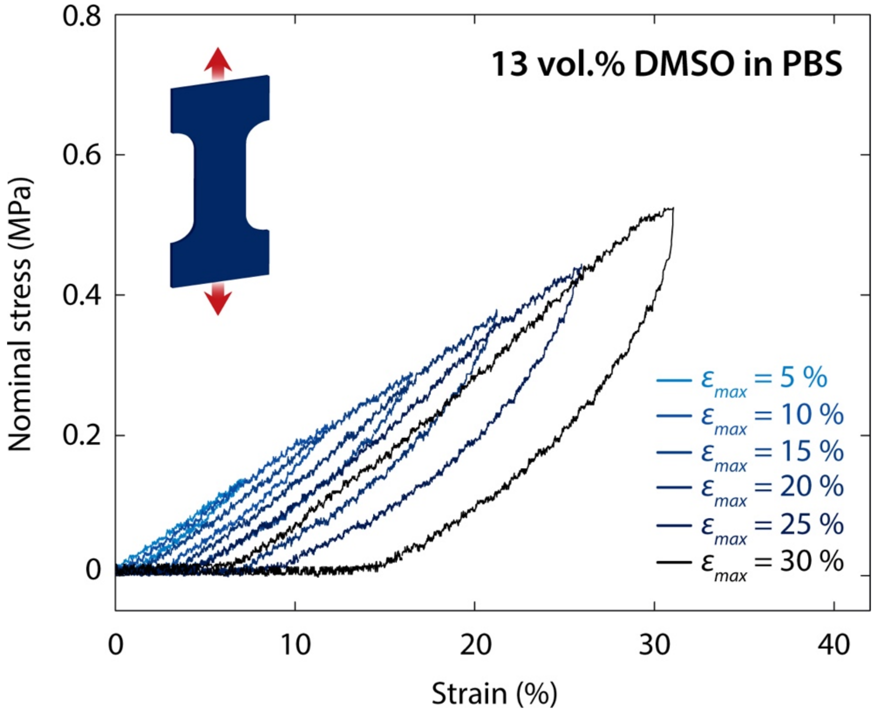

Supplementary Figure 8 | Cyclic tensile deformations of pure PEDOT:PSS hydrogel in PBS. Pure PEDOT:PSS hydrogels based on 13 vol.% DMSO concentration exhibits moderate level of plastic deformation during cyclic tensile deformation from 5 % to 30 %.

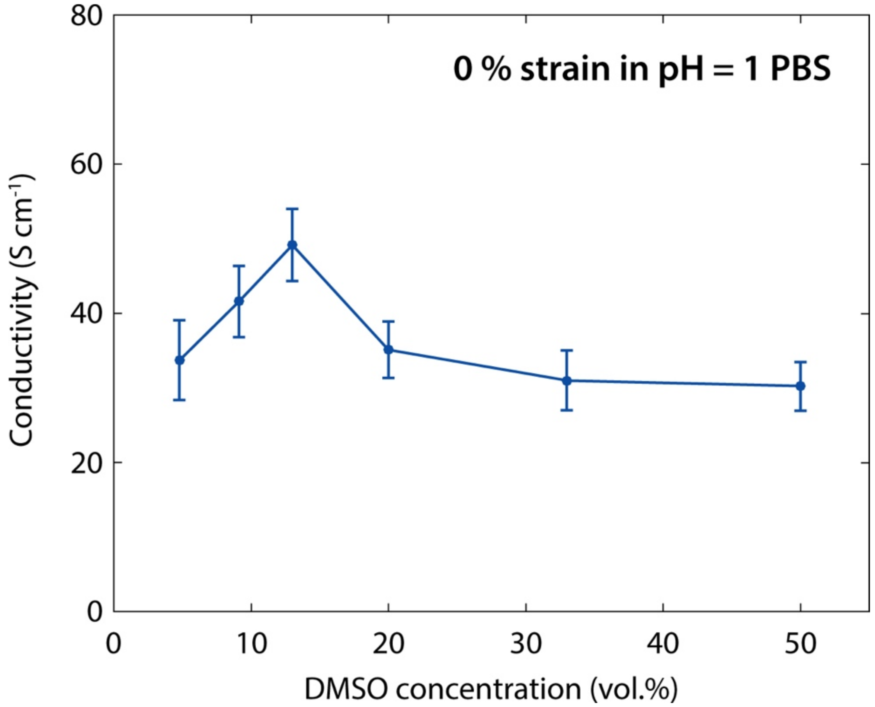

Supplementary Figure 9 | Electrical conductivity of pure PEDOT:PSS hydrogels in acidic PBS. The pH of PBS is adjusted to 1 by adding HCl. Values represent mean and the error bars represent the s.d. of measured values (n = 4).

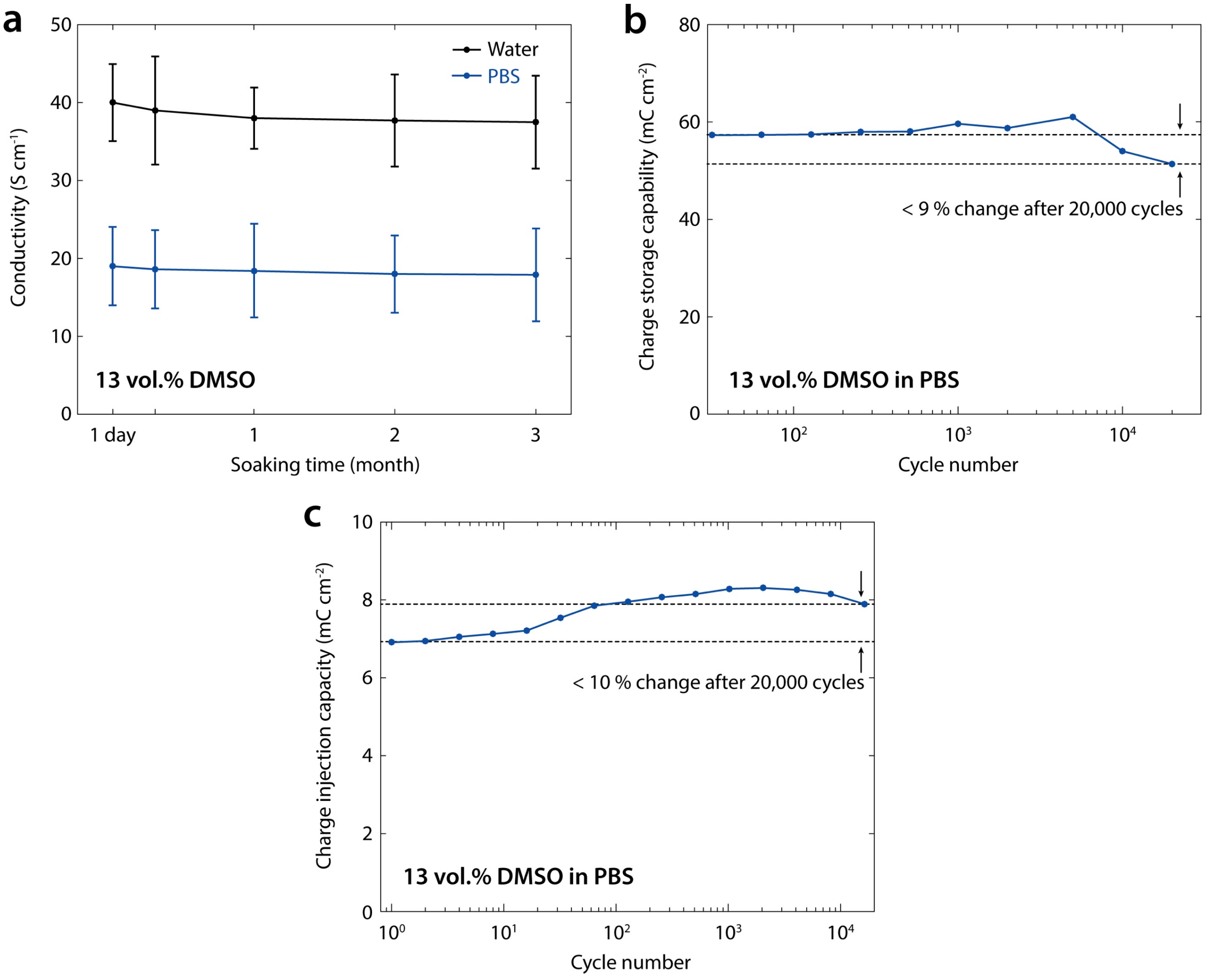

Supplementary Figure 10 | Electrical and electrochemical stability of pure PEDOT:PSS hydrogels in wet environment. (**a**) Electrical conductivity of pure PEDOT:PSS hydrogels exhibit good stability both in PBS and deionized water over 3 months. (**b**) CSC of pure PEDOT:PSS hydrogel shows good stability in PBS with less than 9 % change after 20,000 cycles. (**c**) CIC of pure PEDOT:PSS hydrogel shows good stability in PBS with less than 10 % change after 20,000 cycles. Values in **a** represent mean and the error bars represent the s.d. of measured values (n = 4).

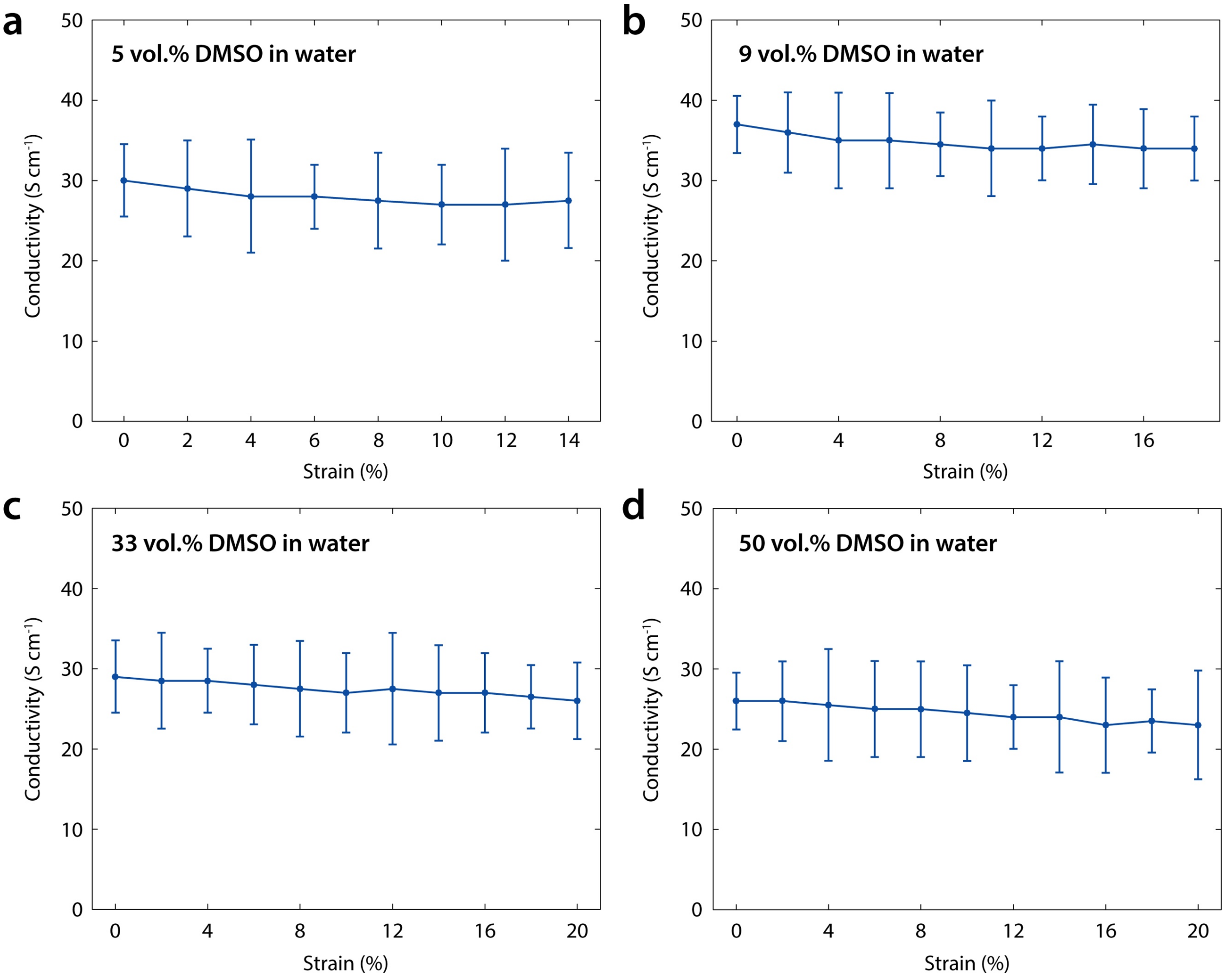

Supplementary Figure 11 | Electrical conductivity of pure PEDOT:PSS hydrogels at different strains in deionized water. (**a-d**) Electrical conductivity of pure PEDOT:PSS hydrogels measured at different tensile strains in deionized water based on (a) 5 vol.% , (b) 9 vol.%, (c) 33 vol.%, and (d) 50 vol.% DMSO. Values in **a-d** represent mean and the error bars represent the s.d. of measured values (*n* = 4).

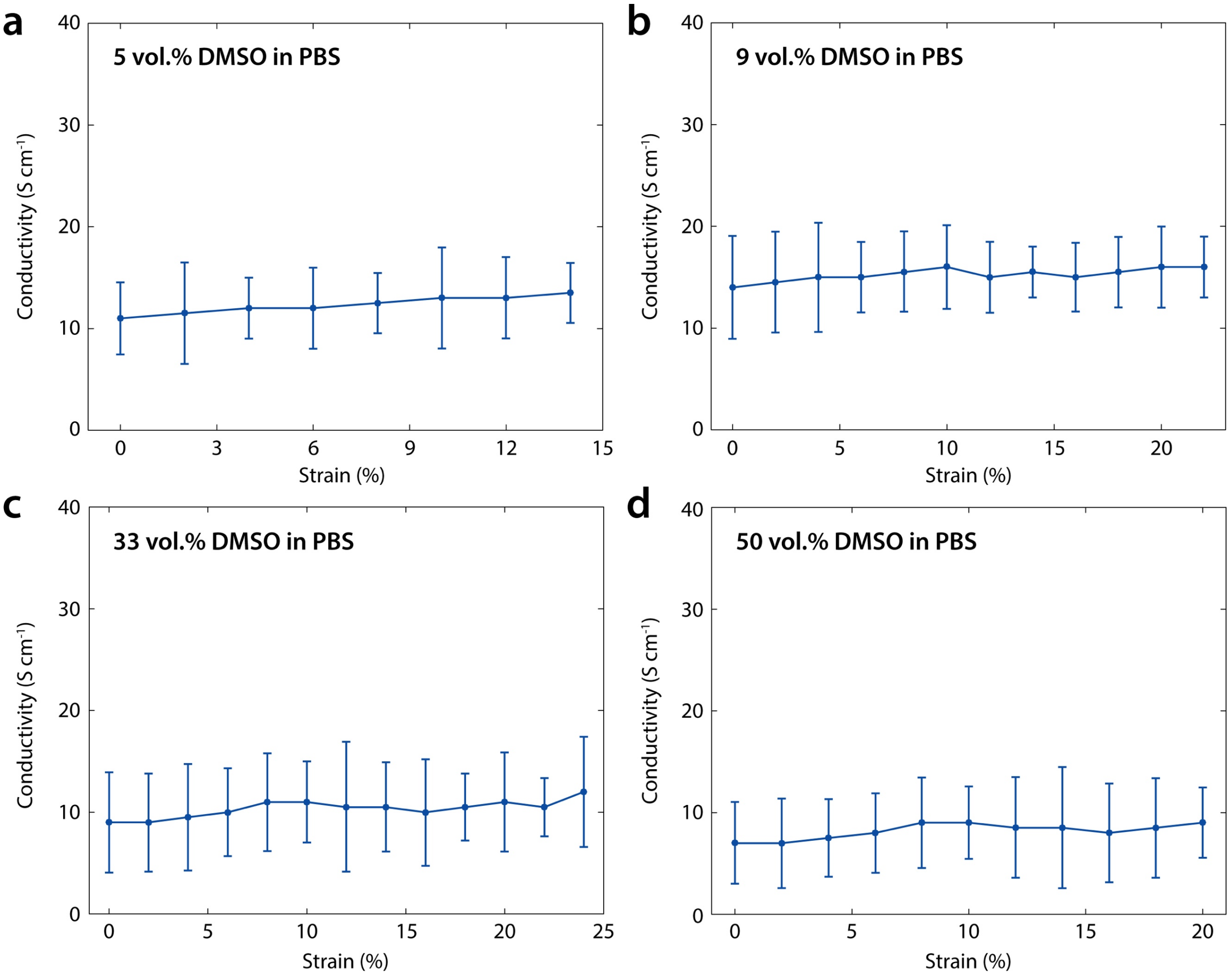

Supplementary Figure 12 | Electrical conductivity of pure PEDOT:PSS hydrogels at different strains in PBS. (**a-d**) Electrical conductivity of pure PEDOT:PSS hydrogels measured at different tensile strains in PBS based on (a) 5 vol.% , (b) 9 vol.%, (c) 33 vol.%, and (d) 50 vol.% DMSO. Values in **a-d** represent mean and the error bars represent the s.d. of measured values (n = 4).

Supplementary Table 1 | Electrical conductivity, measurement condition, and preparation method of various pure conducting polymer hydrogels.[1-6]

Conducting polymer	Conductivity (S cm^{-1})	Measurement condition	Preparation method	Reference
Poly(carboxybetaine thiophene-co-thiophene-3-acetic-acid)	2.7×10^{-7}	Water	Free radical polymerization of custom synthetized macromonomer precursor	*(1)*
Poly(3-thiopheneacetic acid)	$10^{-6} \sim 10^{-3}$	Water	Gelation in DMSO for 2 days followed by solvent exchange to water for 2 days	*(2)*
Polypyrrole	5×10^{-3}	Water	Oxidative polymerization of monomer precursor followed by aging for 30 days	*(3)*
PEDOT:PSS	10^{-2}	Water	Oxidative polymerization of monomer precursor followed by equilibriation in water at least 1 week	*(4)*
Polyaniline	0.23	Water	Oxidative polymerization of monomer precursor followed by purification with water at least1 day	*(5)*
PEDOT:PSS	8.8	Water	Incubation of PEDOT:PSS aqueous solution within sulfuric acid for 3 hours followed by addidtional sulfuric acid treatment for 12 hours	*(6)*
**PEDOT:PSS**	**20 ~ 40**	**Water & PBS**	**Facile dry-annealing of DMSO-added PEDOT:PSS aqueous solution**	**This work**

Supplementary References

1 Cao, B. *et al.* Integrated zwitterionic conjugated poly (carboxybetaine thiophene) as a new biomaterial platform. *Chemical Science* **6**, 782-788 (2015).

2 Mawad, D. *et al.* A single component conducting polymer hydrogel as a scaffold for tissue engineering. *Advanced Functional Materials* **22**, 2692-2699 (2012).

3 Lu, Y. *et al.* Elastic, conductive, polymeric hydrogels and sponges. *Scientific Reports* **4**, 5792 (2014).

4 Dai, T., Shi, Z., Shen, C., Wang, J. & Lu, Y. Self-strengthened conducting polymer hydrogels. *Synthetic Metals* **160**, 1101-1106 (2010).

5 Pan, L. *et al.* Hierarchical nanostructured conducting polymer hydrogel with high electrochemical activity. *Proceedings of the National Academy of Sciences* **109**, 9287-9292 (2012).

6 Yao, B. *et al.* Ultrahigh - Conductivity Polymer Hydrogels with Arbitrary Structures. *Advanced Materials* **29**, 1700974 (2017).
