# Supplementary material for: Pure PEDOT:PSS hydrogels
Source: Nat Commun. 2019 Mar 5;10:1043. doi: 10.1038/s41467-019-09003-5 (PMC6401010; doi:10.1038/s41467-019-09003-5)
Supplement: Supplementary file 2 — Description of Additional Supplementary Files [file 41467_2019_9003_MOESM2_ESM.pdf]

Description of Additional Supplementary Files

File Name: Supplementary Movie 1
Description: Dissociation of dried pristine PEDOT:PSS in PBS.

File Name: Supplementary Movie 2
Description: Isotropic drying of PEDOT:PSS aqueous solution.

File Name: Supplementary Movie 3
Description: Anisotropic drying of PEDOT:PSS aqueous solution on the polypropylene substrate.

File Name: Supplementary Movie 4
Description: Isotropic swelling of pure PEDOT:PSS hydrogel microball prepared from PEDOT:PSS aqueous solution with 5 vol.% DMSO in PBS.

File Name: Supplementary Movie 5
Description: Anisotropic swelling of pure PEDOT:PSS hydrogel film prepared from PEDOT:PSS aqueous solution with 5 vol.% DMSO in PBS.

File Name: Supplementary Movie 6
Description: A free-standing pure PEDOT:PSS hydrogel sample in PBS under tensile stretching until failure.

File Name: Supplementary Movie 7
Description: Anisotropic drying and swelling of patterned pure PEDOT:PSS hydrogel on the polyethylene terephthalate substrate.
